# Gender bias in video game dialogue

**DOI:** 10.1098/rsos.221095

**Published:** 2023-05-24

**Authors:** Stephanie Rennick, Melanie Clinton, Elena Ioannidou, Liana Oh, Charlotte Clooney, E. T., Edward Healy, Seán G. Roberts

**Affiliations:** ^1^ Department of Philosophy, University of Glasgow, Glasgow G12 8QQ, UK; ^2^ Institute of Forensic Linguistics, Aston University, Birmingham B4 7DU, UK; ^3^ School of English, Communication and Philosophy, Cardiff University, Cardiff, UK

**Keywords:** video games, gender, computational linguistics, corpus linguistics

## Abstract

Gender biases in fictional dialogue are well documented in many media. In film, television and books, female characters tend to talk less than male characters, talk to each other less than male characters talk to each other, and have a more limited range of things to say. Identifying these biases is an important step towards addressing them. However, there is a lack of solid data for video games, now one of the major mass media which has the ability to shape conceptions of gender and gender roles. We present the *Video Game Dialogue Corpus*, the first large-scale, consistently coded corpus of video game dialogue, which makes it possible for the first time to measure and monitor gender representation in video game dialogue. It demonstrates that there is half as much dialogue from female characters as from male characters. Some of this is due to a lack of female characters, but there are also biases in who female characters speak to, and what they say. We make suggestions for how game developers can avoid these biases to make more inclusive games.

## 1. Introduction

Studies of the gender distribution of fictional dialogue show that male characters speak more than female characters in films, television shows, radio shows, plays and books [1–9]. This inequality in representation perpetuates stereotypes that can negatively affect audiences’ conceptions of gender and gender roles [10,11]. One important step towards addressing these problems is to quantify the extent of the inequality. However, there are currently no large-scale, systematic studies that quantify this for video games.

This gap in the literature is significant for three reasons. First, video games have become one of the dominant forms of media: in North America, Europe and Asia, video games are played by over half the population and are now played by roughly equal numbers of men and women [12–17]. This makes it an increasingly influential medium that can shape perceptions of gender roles [18]. Secondly, sexist attitudes in video game culture are rife [19–23], with women often being treated as outsiders to the gaming community [24–26]. These attitudes are reflected in the representation of female characters within video games. For example, a number of previous studies demonstrate a systematic under-representation of female characters appearing in games [27–32] as well as related material, including game covers and marketing [33–35]. Many studies have identified that female characters are more likely to be sexualized than male characters [27,29,36–38], and to be positioned in more minor roles [32,39]. The general trend is that female characters appear far less frequently than male characters, and when they do, their representations often reinforce harmful stereotypes [40]. However, debates tend to centre on specific games or characters, with claims susceptible to criticisms of cherry-picking [31,41], or do not directly consider dialogue, which is a significant facet of representation. Finally, an objective study is needed since subjective audience perceptions of the gender distribution of speech are unreliable [42,43].

For all these reasons, objective, large-scale data are required to test whether there is a systematic bias in video game dialogue (where by ‘bias’ we mean an objective, statistical difference from a baseline, as opposed to a measure of people’s intentions). Specifically, we need to obtain three key measures of the distribution in terms of who speaks (and whether this is changing over time), who they speak to, and whether there are differences in what is said. This approach builds on work in ‘ludolinguistics’, the application of linguistic methods to many types of games [44–47] and the discourses that surround them [48–51], particularly the application of corpus linguistic methods to the content of video games [52–57]. However, existing data sources are limited due to non-standardized formats, a limited number of games [58], or a lack of mapping between dialogue and characters [59,60].

To obtain these key measures, we created the *Video Game Dialogue Corpus*, the largest openly available corpus of video game dialogue. This includes 6 million words of dialogue from 13 587 characters from 50 role-playing video games (RPGs [61]) where dialogue is a major game mechanic (as opposed to shooters, beat-em-ups, racing, puzzle, sports or strategy games). It addresses several challenges for obtaining quantitative measures from video game dialogue [60]. Firstly, since the amount of dialogue in games regularly exceeds that of other media (e.g. *Oblivion* has over 700 000 words of dialogue), semi-automated methods are used to parse and analyse the data. Secondly, there are often multiple ways a game can be played such that different dialogue is heard (‘multilinear’ experiences [62–64]). For instance, conversations frequently contain complex, often recursive, choice structures, and may be optional or triggered by various conditions. The corpus format allows the representation of these choice structures, in a format readable by both machines and humans. Thirdly, custom software tools allowed us to correctly assign dialogue to particular characters, and code characters for relevant attributes. All data and software are openly available, allowing replication and expansion. This makes it possible for the first time to measure and monitor gender representation in video game dialogue.

All characters in the corpus have been coded by hand for *conferred gender* [65]. That is, a character’s gender as is likely to be experienced by a typical player. In real life, we can request and defer to individuals’ testimony regarding their gender, whereas game characters only rarely provide such testimony. Additionally, some character features are more reliable indicators of gender in video games than in real life. For instance, some games indicate female characters with bows, the colour pink, or makeup, with the implication that characters lacking such signifiers are male; by contrast, the absence of a bow in real life need not suggest an individual is male. Given these considerations, we use a non-hierarchical list of defeasible indicators when coding gender: we do not assume that any particular indicator takes precedence over another, and each character is coded according to the context of the specific game. Indicators include: character title (e.g. ‘King’), appearance, self-identification or identification by others in dialogue (e.g. pronouns, descriptions), voice actor identity, and information on fan wikis. Our coding system allowed for an open set of possible gender categories; however, most characters belonged to either ‘male’ or ‘female’. Where there was uncertainty, we used contextual information to identify the coding most likely to reflect player experience. If gender was impossible to code, the character was coded as ‘neutral’.

Based on the existing literature, we would predict to observe less female than male dialogue, not necessarily in each individual game, but as a pattern of statistical bias across the medium. Although there are many proposed external reasons for such biases, including perceptions of players, and developer demographics [32,36,66,67], we are focusing on in-game explanations. These might include a lack of female characters overall [68], female characters being relegated to minor roles, and many archetypal character roles being male by default [32,69]. Measures such as the Bechdel test show that there is less likely to be interaction between two female characters in film and television [70,71], and we would expect to see the same pattern in games. We would also expect to see common gender stereotypes reflected in characters’ dialogue [3,72,73]. A growing awareness of gender stereotypes and an increasingly diverse player base who demand better representation in games [74,75] would predict that the proportion of female dialogue increases over the last four decades.

Below, we use the corpus to test these predictions. We identify in-game reasons for these statistical biases and use the data to suggest ways they might be avoided.

## 2. Methods

### 2.1. Sample

Candidate games were identified with the following requirements: the game belongs to the RPG genre; dialogue is a major game mechanic; the game has official English dialogue; the game has sold (or belongs to a series that has sold) at least 1 million copies worldwide; the game frequently appears in lists of the top RPGs of all time. After this, we performed a stratified sample according to a sampling frame with the following criteria: publication date (balanced from 1986 to 2020), RPG style (‘Western’ versus ‘Japanese’ RPGs), and target audience (rated for ‘Everybody’, ‘Teen’ and ‘Adult’ by the Entertainment Software Rating Board). For each candidate, we sought a suitable source of data from a range of sources including data directly from the game code and public websites such as wikis and fan-made transcripts. If none existed, or the source did not contain enough data to reconstruct mappings between characters and dialogue, the candidate was excluded. In order to facilitate diachronic comparison, an attempt was made to source each main entry in some series (e.g. Final Fantasy, King’s Quest, Mass Effect). Sampling proceeded by attempting to balance the sampling frame. See table 1 and electronic supplementary material, S1.2 for more details.

**Table 1.** Sample of games in the corpus. Titles with asterisks indicate multiple games within a series. Numbers in brackets show the number of games in each category. The balance of games over time is not show here, see electronic supplementary material, S1.2.

rating	Western RPG genre	Japanese RPG genre	totals
Everyone	King’s Quest*, Monkey Island*, Stardew Valley (13)	Super Mario RPG, Kingdom Hearts* (5)	18
Teen	Horizon Zero Dawn, Star Wars: Knights of the Old Republic (2)	Chrono Trigger, Final Fantasy* (18)	20
Mature	Mass Effect*, Elder Scrolls*, Dragon Age* (9)	Persona* (3)	12
totals	24	26	50

### 2.2. Script parsing

For each game script, a custom python program was written which scraped and parsed the script into a common format. This parser used systematic pattern recognition, but also applied specific manual edits listed in the metadata files. There were approximately 20 000 manual edits applied to the corpus, mostly fixing mappings between character names and lines of dialogue. The scraping and parsing programs are available in an online repository alongside programs for calculating all the measures and statistics presented in this paper (https://github.com/seannyD/VideoGameDialogueCorpusPublic).

The game script format represented lines of dialogue paired with the name of the character who spoke them, as well as actions and changes in location. The format used a recursive JavaScript object notation (JSON) structure in order to represent dialogue trees common in games.

The game scripts were validated with a systematic error-checking procedure (see electronic supplementary material, S1.2). Transcription errors in the source were identified by finding a video of the game being played, choosing random dialogue in the video, and checking that this dialogue appears accurately in the corpus. Parsing errors from the automatic parsers were identified by manually checking random lines of dialogue. For each game, 15 lines were checked for transcription errors, and five lines were checked for parsing errors. Any errors were raised as issues on the GitHub repository, and fixed. After this, a second round of error checking and fixing was conducted following the same steps as above.

### 2.3. Gender coding

Conferred gender of characters was coded manually according to a set of defeasible indicators, as discussed above and in more detail in electronic supplementary material, S1.3. The coding scheme did not assume binary gender. Evidence for edge cases is documented in the corpus repository. Where there was insufficient evidence for a character’s gender, they were labelled as ‘neutral’ (approx. 7.59% of characters).

To establish the reliability of gender coding, a sample of characters was coded by a secondary coder. For each game, 10 characters were randomly chosen with the probability of being chosen being in proportion to the amount of dialogue they spoke. Agreement between coders was ‘almost perfect’ [76] (raw agreement=96%, Cohen′*s kappa* = 0.92 [0.89, 0.96], see electronic supplementary material, S1.15).

### 2.4. Measures

The measures of dialogue length and readability were obtained using the python module *textatistic* (https://pypi.org/project/textatistic/) that was designed for looking at gender differences in large text corpora [77]. The length of dialogue was measured in number of words, number of lines, number of sentences and number of syllables. All of these measures were correlated with each other with *r* > 0.98 (measured at the group level), indicating that the measures are robust.

Several of the games in the sample were originally written in Japanese, so there may be differences in estimates of female dialogue in the original script versus the English translation. To test this, we analysed three versions of *Chrono Trigger*: the original Japanese script and two English translations. The measures of dialogue length were highly similar between all texts (correlation between number of English words and Japanese characters per line *r* = 0.93) and all estimates of the proportion of female dialogue are within 0.7 percentage points of each other (see electronic supplementary material, S1.13). This suggests that the general gender biases are not caused by translation.

### 2.5. Statistical methods

The aim of the statistical measures is to assess whether there is a statistically significant bias in the distribution of dialogue by gender within a game. Standard parametric tests are inappropriate because the data is highly non-independent (words belong to lines, and lines belong to coherent characters) and highly skewed (a small number of characters say a lot while a large number of characters say little). Instead, a permutation framework was used that compares an observed measure with the range of measures that would be expected if there really was no bias. This was done as follows (for more details, see electronic supplementary material, S1.4).

The proportion of dialogue by gender is assessed in comparison with two baselines which reflect two possible sources of bias. The first source is a ‘character bias’ where more male characters are included in the game than female characters, which has a knock-on effect on the proportion of dialogue for each gender. A hypothetical script was generated where the mapping between gender categories and individuals is randomly determined, with each character having an independent and equal probability of being male or female. That is, the link between gender and characters is randomized to remove any potential bias. Generating 100 000 scripts created a distribution of probable values for the proportion of female dialogue if there was no bias. This was compared with the true proportion of female dialogue. This produced a *z*-score that represents the strength of the bias (in number of standard deviations away from the expected mean), and a *p*-value that represents how likely the baseline process results in a measure that is more extreme than the observed distribution. Lower *p*-values indicate that the baseline model assumptions are unlikely to hold, and suggests that the imbalance in dialogue is due to the imbalance in the proportion of characters.

Similarly, the second possible source of bias is the ‘dialogue bias’ where the average male character is given more dialogue than the average female character. To model a baseline to test this, a hypothetical script is generated where the mapping between gender and characters remains unchanged, but the mapping between characters and lines is randomly permuted (all of one character’s lines can be swapped for all of another character’s lines). This maintains the same proportion of female characters as the real data, but the proportion of dialogue for female characters will vary if they are given systematically less to say than male characters. A total of 100 000 scripts were generated to create a distribution of values, with which the true proportion of female dialogue can be compared.

To assess imbalances in who speaks to whom, we also used a permutation method to establish baselines. To assess the effect of player choice, we simulated a player making random choices and making optimal choices to maximize either male or female dialogue. These, and all other statistical tests are described in the electronic supplementary material.

### 2.6. Survey

In order to compare the objective measures with player perceptions of gender balance, an online survey was conducted. One hundred and eighty-eight adult participants were recruited via online platforms related to gaming and answered questions about their subjective estimations of gender balance in RPGs. This included the average percentage of words spoken by female compared with male characters, and the percentage of games where female characters had more dialogue than male characters. The reference gender of the questions was randomized between participants (see electronic supplementary material, S1.6).

### 2.7. Qualitative methods

The data for certain games facilitated qualitative analysis of specific games, which helped assess biases in the content of what characters say and what they use language to do. A five-step thematic analysis [78] was applied to compare the dialogue of the character of Jesse between *Final Fantasy VII* (released in 1997) and *Final Fantasy VII: Remake* (released in 2020, a modern ‘remake’ with the same characters, setting and plot; see electronic supplementary material, S1.10). The quest structure of *Daggerfall* was used to compare the recruitment strategies of male and female characters using critical discourse analysis [79] (see electronic supplementary material, S1.11). The choice structure for *Stardew Valley* allowed the differences in dialogue according to the player character gender using critical content analysis ([80]; see electronic supplementary material, S1.12). Finally, a parallel corpus of dialogue from *Chrono Trigger* in English and Japanese allowed us to check the influence of translation on gender representation using methods from translation studies ([81,82]; see electronic supplementary material, S1.13).

## 3. Results

The final corpus comprised 6 280 892 words of dialogue. We use quantitative and qualitative methods to study who speaks, who they speak to and what they say.

### 3.1. Who speaks?

Thirty characters (0.25%) were coded as belonging to gender categories other than male or female (e.g. Quina Quen from *Final Fantasy IX* is described as genderless), which was too few to study systematically. There were 5 679 321 words (404 609 lines) spoken by characters coded as either male or female. Of those, 35.16% were spoken by female characters. This is significantly lower than would be expected if the gender of each character was assigned randomly to either male or female (permutation *z* = 9.52, *p* = 0.00001). However, female characters are not systematically given fewer words of dialogue *per character* than male characters (permutation *z* = −1.24, *p* = 0.890). The overall imbalance is more likely to be due to there being significantly fewer female characters than male characters (29.37% female, 70.63% male, binomial test *p* < 0.0001).

**Figure 1.**
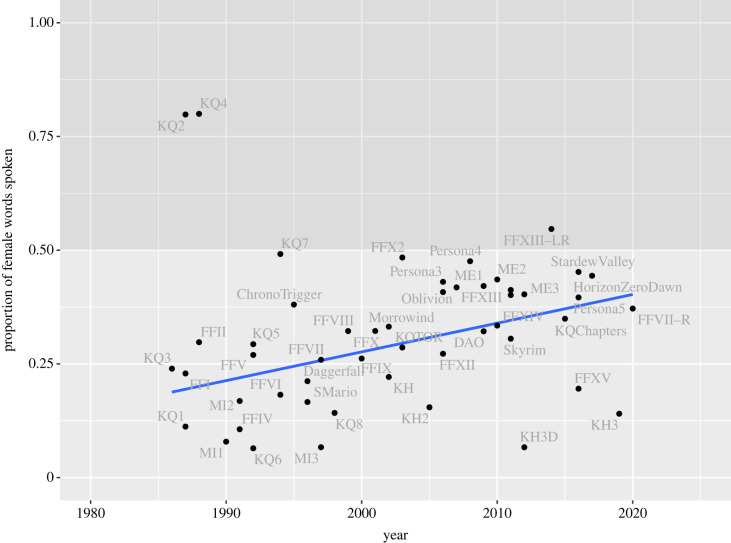
Proportion of female dialogue compared with male dialogue in 50 video games over time. Acrynoms refer to game series (FF, Final Fantasy; KQ, King′*s Quest*; KH, Kingdom Hearts; MI, Monkey Island; ME, Mass Effect; DA, Dragon Age; KOTOR, Star Wars: Knights of the Old Republic). The regression line shows linear change, excluding two outliers (KQ2, KQ4).

**Figure 2.**
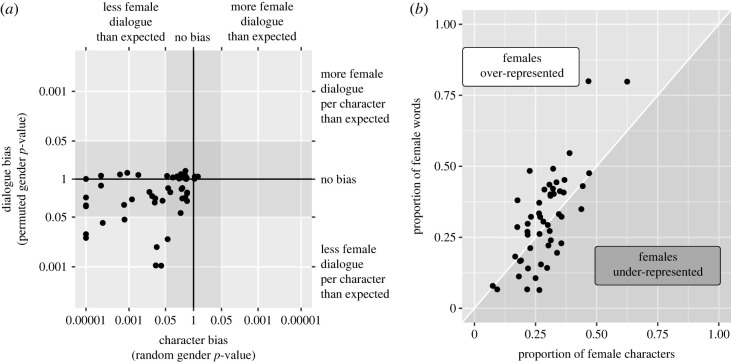
(*a*) Estimations of gender bias in 50 video games. The horizontal axis represents the extent of the character bias: the likelihood of the observed dialogue balance being generated if characters were assigned gender randomly. The vertical axis represents the extent of the dialogue bias: the likelihood of the observed dialogue balance being generated if character gender was permuted. Values further from the centre indicate more extreme results. (*b*) The proportion of words spoken by female characters in each game as a function of the proportion of female characters. The space is split at the identity (intercept 0 and a slope of 1) to show games where female dialogue is over-represented given the proportion of characters, and games where the female dialogue is under-represented given the proportion of characters.

In individual games, the proportion of female dialogue ranged from 6% (*King’s Quest VI*) to 80% (*King’s Quest IV: The Perils of Rosella*) (figure 1). Only three games had more than 50% female dialogue (*King’s Quest II*, *King’s Quest IV* and *Lightning Returns: Final Fantasy XIII*). Twenty-eight games (46%) had significantly less female dialogue than would be expected if gender was assigned randomly (figure 2). Ten of these games also had significantly less female dialogue than expected even when taking the proportion of characters into account. No games had significantly more female dialogue than male dialogue compared with either baseline, including games with multiple female protagonists (*Final Fantasy X-2*, *King’s Quest VII*).

However, the bias is not just due to the dialogue of major characters (see electronic supplementary material, S1.5). In fact, the bias is actually more extreme for minor characters. The proportion of female dialogue is lower for the least talkative quarter of characters in each game (31% female dialogue) compared with the most talkative quarter (36% female dialogue, *χ*^2^ = 16 466.8, *p* = 0.0001), despite no significant difference in the proportions of characters in each gender in each quarter (*χ*^2^ = 3.28, *p* = 0.35).

Some of these results are not surprising to players. In a survey of 188 video game players (see electronic supplementary material, S1.6), the average guess of the percentage of female dialogue was not significantly different from the empirical estimate (average guess=31%, *t* = −0.68, *p* = 0.4964). However, on average, players overestimated the percentage of games that would have more than 50% female dialogue (average guess 24%, *t* = 10.47, *p* < 0.0001). That is, although players anticipated the general trend towards more male dialogue, they overestimated the number of exceptions to that trend.

Analyses of individual games revealed further possible in-game explanations for the difference in the amount of dialogue uttered by female or male characters. For instance, we found evidence to support the prediction that female characters are relegated to a smaller number of roles. In *Elder Scrolls: Oblivion*, the game code assigns each line of dialogue with one particular emotion (anger, disgust, fear, happiness, sadness, surprise or ‘neutral’) and intensity (see electronic supplementary material, S1.7). Female non-player characters (NPCs) are more likely to have dialogue paired with neutral emotions (*χ*^2^(1) = 34.6, *p* < 0.0001) and to have default intensity (*χ*^2^(3) = 69.97, *p* < 0.0001). We also found that generic male NPCs are more than four times more likely to be given unique lines of dialogue than generic female NPCs (*χ*^2^(1) = 365.91, *p* = 0.0001).

Some biases are encoded into the game’s algorithms. In *Daggerfall*, the distribution of male and female characters is determined randomly. However, before being assigned a gender, NPCs are first assigned a role and if they are a guard, they are male by default.

Dialogue in games can vary based on player choices, but this does not always have much effect on gender representation. For example, 11 games in our sample allow the player to choose the gender of their player character. Of these, only the Mass Effect trilogy and Dragon Age 2 have more than 50% female dialogue when the player character was female, and these are only just over equal proportions (the highest being for Dragon Age 2 with 66% compared with 22% female dialogue if the player character is male).

**Figure 3.**
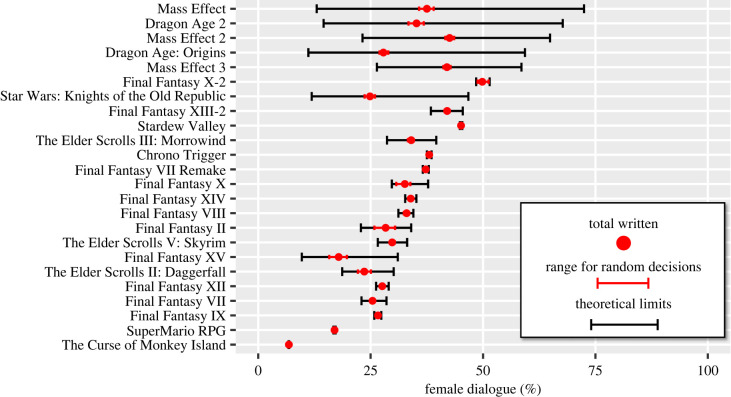
How the proportion of female dialogue varies according to player choices in dialogue trees. For each game, the figure shows: the proportion of female dialogue written by the game authors (red dot); the range of proportions from a player making random decisions (red whiskers); and the range from the proportion of female dialogue experienced if an omniscient player tried to maximize male dialogue to if they are trying to maximize female dialogue (black whiskers).

Another source of variation is the choice in dialogue trees. For 24 games where dialogue tree information was available, we simulated an omniscient player who tries to maximize dialogue from one gender while minimizing the dialogue from another (without revisiting lines, figure 3). A player trying to maximize female dialogue over male would succeed in observing more female dialogue than male dialogue in 35.7% of dialogue trees, on average seeing 10.2 more words spoken by females than males in each dialogue tree. By contrast, when maximizing male dialogue over female, a player would succeed in observing more male dialogue than female in 64.6% of dialogue trees, on average seeing 33.4 more words from males than females (significantly greater than for maximizing female dialogue, *t* = 59.71, *p* < 0.001). This suggests that the bias against female dialogue cannot be easily avoided by players.

There was a positive correlation between a game’s year of publication and the proportion of female dialogue (*r* = 0.47 [0.22, 0.67], *n* = 50, *p* = 0.001). From 1986 to 2020, female dialogue increased by 6.3 percentage points per decade. The weak positive trend aligned with most player’s perceptions in the survey responses. Were this rate to continue, gender balance would not be reached until 2036.

### 3.2. Who speaks to whom?

Previous studies of film have demonstrated a lack of dialogue between female characters, commonly discussed in reference to the *Bechdel test* which is passed only if a work includes two named female characters who talk to each other about something other than a man [70]. Some games in the corpus fail this test (e.g. the *Monkey Island* games, which only have five female characters across the three games). However, this test can be passed with a single line of dialogue, so it does not provide nuanced information about the extent of biases [83]. We take a statistical approach, where the proportions of transitions between characters of different genders is compared with a baseline of expected proportions if the lines of dialogue were placed in a random order.

We found that there is a significant bias for men to be followed by men, and against women being followed by women, even accounting for the greater proportion of male characters: for 38 games where dialogue order was available, all showed some significant bias in transitions (see electronic supplementary material, S1.9). In the whole corpus, a male is followed by another male in 66% of transitions. This is 10 percentage points higher than what would be expected if the lines of dialogue were in a random order (*z* = 10.59, *p* = 0.0001). All but 5 of the 38 games exhibit this bias. Conversely, 32% of the time, a female is followed by a female, which is 11 percentage points lower than would be expected if lines were in a random order (*z* = −9.11, *p* < 0.001). All but seven games exhibit this bias.

This bias can be detected even on top of a general bias against female dialogue. For example, in Final Fantasy XV, which centres four male playable characters and has only 20% female dialogue, the bias against females talking to females is still observed, even controlling for the number of male lines of dialogue (male-to-male *p* < 0.001, female-to-female *p* < 0.001). Conversely, in Final Fantasy X-2, which centres three female playable characters, the proportion of female-to-female transitions is not different from expected (*z* = 1.17, *p* = 0.126) and the proportion of male-to-male transitions is lower than expected (*z* = −3.58, *p* < 0.001).

### 3.3. What is said?

We have demonstrated that there are gender biases in terms of how much and to whom characters speak. But representation also involves the content and function of what characters say. In line with some other studies of gendered language [84–86], we found that, compared with male characters, female characters display more gratitude (+32%, *G*2 = 145.03, *p* < 0.001), use more hedging (+18%, *G*2 = 185.08, *p* < 0.001), apologise more (+29%, *G*2 = 82.9, *p* < 0.001), and swear less (−37%, *G*2 = 414.06, *p* < 0.001). These are all markers of greater politeness [87,88] which reflect folk linguistic myths about gendered language that are not robustly supported by empirical studies of real conversation ([89,90], see electronic supplementary material, S1.14).

Several qualitative studies of the corpus also revealed gender biases. For example, giving a character more lines does not necessarily result in better representation. In *Final Fantasy VII*, female character Jessie has 32 lines of dialogue. In the 2020 remake, she has 301. A thematic analysis (see electronic supplementary material, S1.10) revealed three main functions of her dialogue in the original game: demonstrating ability and technical competence (22%), dispensing information (22%) and revealing personality (56%). In the remake, there was less demonstrating competence (10%), less dispensing information (8%) and much more personality revealing (82%), most of which was flirting with the main male character.

There can also be gendered differences in terms of how NPCs use language to motivate the player to act. In *The Elder Scrolls: Daggerfall*, female quest-givers focus on motivations relating to family and relationships, and use stereotypically female linguistic strategies for negotiation (tag questions and hedges, see electronic supplementary material, S1.11).

Another context in which gender biases can arise is when NPCs alter their responses according to the gender the player has chosen for their player character. For example, in *Stardew Valley*, NPCs sometimes respond differently to male and female player characters. Twenty-four per cent of these cases perpetuated a gender stereotype, while there were no cases of subverting a gender stereotype. For example, female players are offered a salad, wine, repeatedly described as beautiful and assumed to have little experience of video games, while male players are offered pasta, ale, described as ‘full of energy’ and are assumed to be good video game players (see electronic supplementary material, S1.12).

## 4. Discussion

Using a unique corpus of video game dialogue, we were able to extract several novel quantitative observations about gender biases. We found that there is almost twice as much dialogue from male characters as from female characters in video games. While this overall bias is not surprising to players, there are also very few games with more female dialogue than male dialogue, even when the main characters are female. There is also an imbalance in who speaks to whom, with female characters less likely to talk to other female characters. These biases cannot be easily avoided by player choices, and there are clear differences in the content of male and female dialogue that align with stereotypes and gendered tropes. We presented evidence for two main causes of this bias: firstly, that there is an imbalance in the proportion of female characters, with male characters being twice as numerous as female characters in nearly three-quarters of games. Secondly, female characters are given a narrower range of roles than male characters. While the average proportion of female dialogue has increased over the last three decades, this change is slow. Even in the last 6 years, two games in the corpus were released with less than a quarter of dialogue spoken by females (*Kingdom Hearts 3*, *Final Fantasy XV*).

More diverse representation in video game content is being called for by players and developers [74,75]. If developers want to improve the representation of female dialogue in games, then the findings above suggest various approaches which might facilitate this. The first implication of our work is that content creation should be monitored during game development. The results here suggest that monitoring all characters, not just main characters, is required, and an aggregate approach using statistical baselines is necessary to understand the patterns. This is an alternative to recent attempts at tools that measure ‘diversity’ of individual (main) characters based on perceived difference from some assumed norm [91], which have received criticism from players and developers [92]. A second potential approach is to use ‘gender flipping’: developing a character as one gender, but then changing their gender signifiers before release. The idea is that this process subverts any subliminal biases about behaviour or roles. Gender flipping is known to have occurred for various reasons in existing games (e.g. Fang from *Final Fantasy XIII*). A third approach might be to use balanced procedural generation of characters, which may be particularly tempting for large-scale, open-world games. While it might seem like this guarantees balance, it is only as good as the algorithms that go into the process. For example, while the algorithm for choosing the gender of ordinary citizens in *Daggerfall* is equally likely to assign male or female gender, it first decides whether the character has a guard’s job, and if so assigns a male gender. This leads to exclusively males in these archetypal positions of authority and slightly more male than female characters in the world. Moreover, procedural creation of dialogue that reflects a coherent discourse and character is still a technical challenge, and reliance on machine-learning methods is likely to simply reflect the biases that are present in the training data [93–95].

These three approaches may help, but might be time-consuming, present technical challenges, or just move responsibility onto ‘black box’ algorithms that are harder to analyse. A simpler solution is suggested by one key insight from this study: that the low proportion of female dialogue reflects the low proportion of female characters. Therefore, the simplest approach for increasing the proportion of female dialogue is to just increase the proportion of female characters in a game. After that, our study suggests that the range of roles for female characters should be increased and the distribution of who talks to whom should be balanced. Developers can monitor these aspects with automated proxy measures such as the ones used in this study (dialogue tree structures, face animation cues, consecutive line transitions, methods from corpus linguistics and NLP) and others available in the game development data (e.g. gameplay data, character model data).

Of course, the proportion of dialogue itself is only a proxy measure for underlying biases in the content of dialogue related to gendered roles and stereotypes. An important future step for research into gender biases in video games is to identify the tropes that result in the imbalances in dialogue so they can be directly addressed. Future research could also look beyond gender to other aspects of representation as well as the intersections between them. Both of these research aims can be facilitated by the data in the *Video Game Dialogue Corpus*.

While we would expect variation in terms of diversity and representation between individual games, what we have shown here are systematic biases across games. Developers concerned about such biases should monitor and reflect on these patterns. This is a perhaps a bigger challenge for video games than any other medium, since modern games can incorporate many more hours of content than the average film or television show, and include hundreds of characters, designed by large numbers of developers, across multiple companies. Monitoring bias on this scale will require automated systems and an understanding of norms in current games. Corpora of dialogue can be part of those systems, enabling developers to make informed choices. To facilitate this the *Video Game Dialogue Corpus* presented in this study is public, open source and expandable (see https://github.com/seannyD/VideoGamesDialogueCorpusPublic).

## Data Availability

All data and scripts for processing and analysing are available in an open source repository: https://github.com/seannyD/VideoGameDialogueCorpusPublic; The supporting information documents all analyses. The data are provided in electronic supplementary material [96].
